# GEC-derived SFRP5 Inhibits Wnt5a-Induced Macrophage Chemotaxis and Activation

**DOI:** 10.1371/journal.pone.0085058

**Published:** 2014-01-08

**Authors:** Chenghai Zhao, Xianmin Bu, Wei Wang, Tingxian Ma, Haiying Ma

**Affiliations:** 1 Department of Pathophysiology, College of Basic Medical Science, China Medical University, Shenyang, China; 2 Department of General Surgery, Shengjing Hospital, China Medical University, Shenyang, China; Centro di Riferimento Oncologico, IRCCS National Cancer Institute, Italy

## Abstract

Aberrant macrophage infiltration and activation has been implicated in gastric inflammation and carcinogenesis. Overexpression of Wnt5a and downregulation of SFRP5, a Wnt5a antagonist, were both observed in gastric cancers recently. This study attempted to explore whether Wnt5a/SFRP5 axis was involved in macrophage chemotaxis and activation. It was found that both Wnt5a transfection and recombinant Wnt5a (rWnt5a) treatment upregulated CCL2 expression in macrophages, involving JNK and NFκB signals. Conditioned medium from Wnt5a-treated macrophages promoted macrophage chemotaxis mainly dependent on CCL2. SFRP5 from gastric epithelial cells (GECs) inhibited Wnt5a-induced CCL2 expression and macrophage chemotaxis. In addition, Wnt5a treatment stimulated macrophages to produce inflammatory cytokines and COX-2/PGE_2_, which was also suppressed by SFRP5 from GECs. These results demonstrate that Wnt5a induces macrophage chemotaxis and activation, which can be blocked by GEC-derived SFRP5, suggesting that Wnt5a overproduction and SFRP5 deficiency in gastric mucosa may together play an important role in gastric inflammation and carcinogenesis.

## Introduction

It is widely accepted that aberrant macrophage infiltration and activation plays a role in tumor initiation. The attribution of macrophage infiltration and activation to carcinogenesis may be partially related to inflammation which has been recognized as a hallmark of cancer [1]–[3]. As an important linker between inflammation and cancer, macrophage releases cytokines, COX-2/PGE_2_, nitric oxide synthases, and chemokines which further chemoattract macrophages and other inflammatory cells into local tissues. Macrophage infiltration was observed in both H.pylori-related gastric inflammation and gastric cancer, which was associated with overproduction of CCL2 (also named MCP-1, monocyte chemotactic protein-1), a chemokine involved in macrophage recruitment in cancer tissues[4], [5].

As a member of noncanonical Wnt family, Wnt5a has been implicated in human malignancies [6]–[8], including gastric cancer [9]. However, the underlying mechanisms of Wnt5a overexpression in these tumors remain largely unknown. Several studies revealed that inflammatory stimuli, such as LPS [10], mycobacteria [11], P. gingivalis [12], induced Wnt5a expression by macrophages. In addition, we previously found that H.pylori also stimulated macrophages to produce Wnt5a which exerted effects on gastric epithelial cells in a paracrinal manner [13]. As Wnt5a receptor Frizzled 5 has been shown to be expressed in macrophages, it is conceivable that macrophage-released Wnt5a can affect macrophages in an autocrinal manner [11].

Secreted Frizzled-Related Proteins (SFRPs) are supposed to antagonize Wnt signaling owing to the harboring of cysteine-rich domains (CRDs). Via CRDs, SFRPs compete with Wnt receptor Frizzled proteins for combining Wnt molecules. SFRP5 is the fifth member of SFRPs family, which has been observed to suppress Wnt5a signaling in addipose tissue [14]. Our previous study revealed that SFRP5 derived from gastric epithelial cells (GECs) inhibited Wnt5a-induced GEC migration [13]. SFRP5 expression was frequently downregulated in gastric cancer due to SFRP5 gene hypermethylation [13], [15], which was hypothesized to lead to uncontrolled activation of Wnt5a signal pathways.

In the present study, we investigated the expression of CCL2 in Wnt5a-treated macrophages to explore the effects of Wnt5a on macrophage recruitment. We also attempted to determine the involvement of Wnt5a in macrophage activation by analyzing the production of inflammatory cytokines and COX-2/PGE_2_. Finally, we evaluated whether GEC-derived SFRP5 could block Wnt5a-induced macrophage chemotaxis and activation, to discover the defensive role of SFRP5 in gastric inflammation and carcinogenesis.

## Methods

### Ethic Statement

This study and experimental protocols involved in human peripheral blood were approved by Biomedical Research Ethics Committee of China Medical University, and written informed consents from the donors were obtained.

### Cells

Human gastric cancer cell BGC-803 [13] and non-cancerous gastric epithelial cell GES-1 were cultured in RPMI 1640 supplemented with 10% fetal bovine serum, at 37°C in a humid incubator with 5% CO_2_. Mononuclear cells were isolated from human peripheral blood [13] by Ficoll-Hypaque density gradient centrifugation, and purified by CD14 microbeads. Monocytes were cultivated in RPMI 1640 supplemented with 10% fetal calf serum for 7–10 days until differentiation into macrophages.

### Real-time PCR

RNA was isolated from cells using Trizol (Takara, Dalian, China) according to the manufacturer’s protocol. 1 µg RNA was reverse transcribed into cDNA using Avian Myeloblastosis Virus (AMV) reverse transcriptase. Real-time PCR was carried out using LightCycler DNA Master SYBR Green I Kit (Roche Diagnostics) in the LightCycler system (Roche Diagnostics). The housekeeping gene GAPDH was used as an internal control. Gene expression was quantified by the comparative CT method, normalizing CT values to GAPDH and calculating relative expression values. Primer sequences for IL-1β, IL-6, TNF-α and GAPDH were described in [16], and for CCL2, CCL5, CCL7 and COX-2 in [5].

### Western Blot

A total of 20 µg protein of each sample was run on a 12% sodium dodecyl sulfate (SDS)/acrylamide gel, and transferred to a 0.2 µm nitrocellucose membranes, which were blocked overnight (4°C in PBS with 0.1% Tween and 10% milk powder). The membranes were then incubated with antibodies. Primary antibodies for Wnt5a, JNK, p-JNK, p65, p-pNFκB65 (p-p65), CCL2, β-actin and the corresponding secondary antibodies were provided by Santa Cruz. Antibody for SFRP5 was purchased from Abcam. Antibodies for CCR2 and CCR4 were obtained from BOSTER (Wuhan, China). The human gene β-actin was employed as an internal control.

### ELISA

Quantikine ELISA kits (Boster, Wuhan, China) were used to determine the concentrations of CCL2, CCL5, CCL7, TNF-α, IL-1β, IL-6 and PGE_2_ in cell (2×10^5^) culture supernatants according to the manufacturer’s instructions. The detection limit of the assay was 2 pg/ml for PGE_2_ and TNF-α, 4 pg/ml for CCL2, CCL5, CCL7, IL-1β and IL-6.

### Immunofluorescence

After incubation of 12 h, macrophages were fixed by 4% (w/v) formaldehyde solution for 15 min and washed with PBS, and further lysed with 0.2% Triton X-100 (Biochemicals) in sterile distilled water. Fixed macrophages were sequentially labeled with rabbit anti-CCL2 antibody (Santa Cruz) and rhodamine-conjugated goat anti-rabbit IgG (Santa Cruz) and observed by fluorescent confocal microscopy.

### Expression Vector Transfection

The pcDNA3.1 (Invitrogen, Paisley, United Kingdom) expression vector was made by cloning of the full-length PCR product of Wnt5a or SFRP5 with PFU DNA polymerase (Invitrogen, Paisley, United Kingdom). Cells were seeded in a 24-well plate, and transfected with 2 µg pcDNA3.1 expression vector or 2 µg pcDNA3.1 empty vector per well at 70% confluence using Lipofectamine 2000 reagent (Invitrogen, Paisley, United Kingdom) according to the manufacturer’s protocol.

### RNA Interference

RNA interference was carried out using Lipofectamine (Invitrogen, United Kingdom) according to the manufacturer’s instructions. Briefly, cells in a 24-well plate were transfected with 50 nM SFRP5 siRNA plasmid at 70% confluence using Lipofectamine 2000 reagent. Nontargeting siRNA plasmid with an identical concentration was used as control. Cells were analyzed 48 hours after transfection. Sequence for CCL2 siRNA was 5′-TGTGAAACATTATGCCTTA-3′, and for SFRP5 in [15].

### Macrophage Chemotaxis Assay

A modified Boyden chamber (24-well, 8 µm pore) was used to assess macrophage chemotaxis. Macrophages (2×10^6^) were added to the upper chamber and conditioned medium was added to the lower chamber. Migration assays were incubated for 8 hours at 37°C and 5% CO_2_. The invasion assay was analyzed with the chamber coated in Matrigel. Migrated or invaded macrophages were fixed and stained with crystal violet according to the manufacturer’s protocol. For each transwell, the number of migrated macrophages in five random microscopy fields was counted.

### Statistical Analysis

Data among groups were compared using Student’s *t*-test or one way ANOVA. SPSS version 11.0 (SPSS, Chicago, IL, USA) was used to carry out statistical analysis. When *P*-value was less than 0.05, difference was considered significant.

## Results

### Wnt5a Upregulates CCL2 Expression in Macrophages

**Figure 1:**
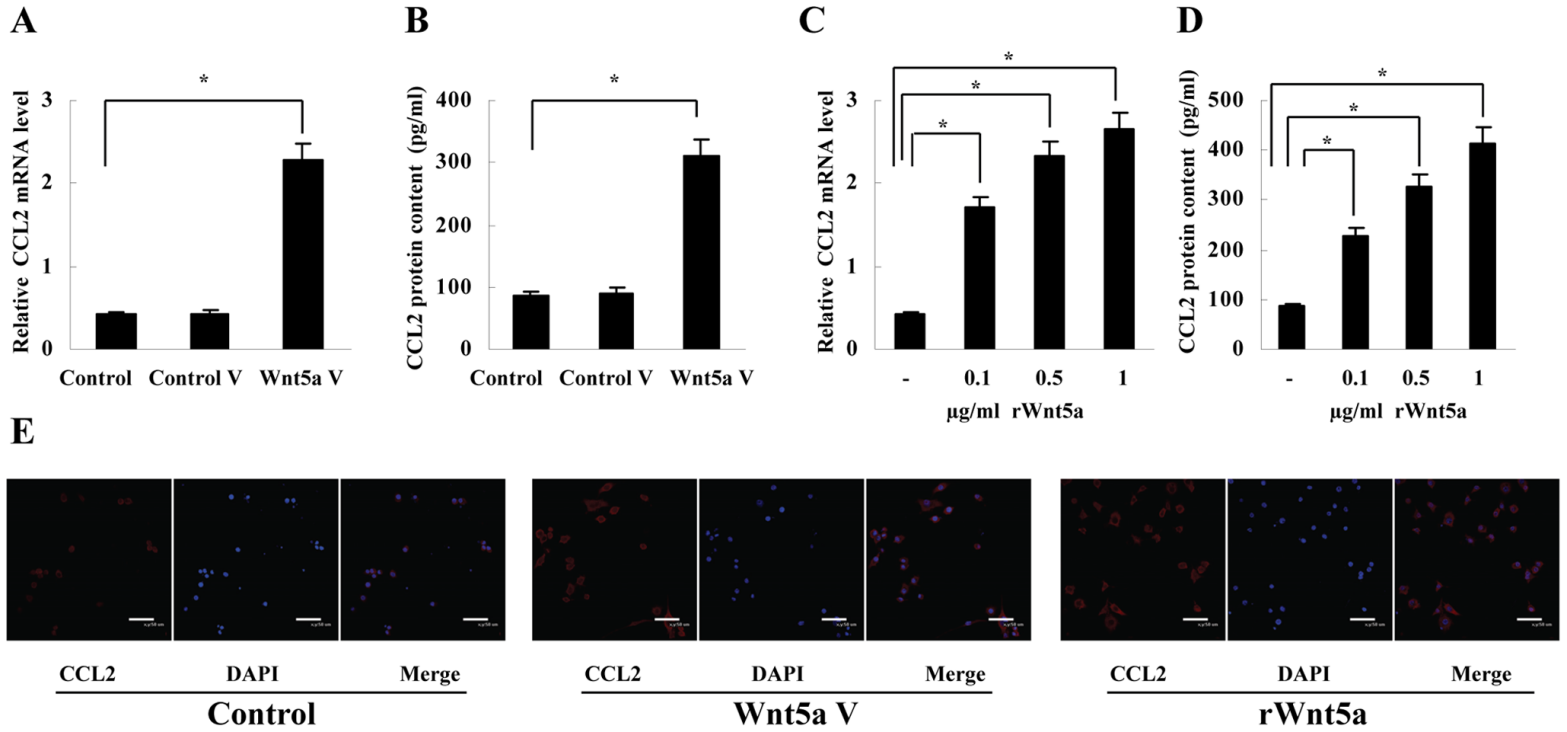
CCL2 upregulation by Wnt5a in macrophages. (A) and (B) Real-time PCR and ELISA showed that CCL2 mRNA expression and protein secretion was upregulated by transfection with Wnt5a expression vector, *P<0.01. (C) and (D) Real-time PCR and ELISA showed that CCL2 mRNA expression and protein secretion was stimulated by rWnt5a (0.1 µg/ml, 0.5 µg/ml, 1 µg/ml, respectively), *P<0.01. (E) Immunofluorescence showed that both Wnt5a transfection and rWnt5a treatment increased CCL2 production. Control V, transfection with control vector; Wnt5a V, transfection with Wnt5a expression vector. Data are expressed as mean±SD, n = 3.

To evaluate whether Wnt5a was engaged in macrophage chemotacxis, we investigated the effect of Wnt5a on the expression of CCL2 which was involved in macrophage recruitment in gastric tumor [5]. After transfection with Wnt5a expression vector (pcDNA3.1-Wnt5a) for 48 hours, macrophages were found to express more CCL2 mRNA and secreted more CCL2 protein (5.4 fold and 3.6 fold, respectively, Figure 1A, 1B; Figure S1A). In addition, we assessed whether Wnt5a transfection induced the expression of CCL2 receptors, CCR2 and CCR4. Unlike CCL2, Western blot detection showed that neither CCR2 nor CCR4 was upregulated by Wnt5a transfection (Figure S1B). To verify the role of Wnt5a in CCL2 induction, we next treated macrophages with recombinant Wnt5a (rWnt5a, R&D system). It was found that rWnt5a treatment for 12 hours had the strongest effect on CCL2 expression (Figure S2), and CCL2 expression was upregulated up to 6.2 fold in mRNA level and 4.8 fold in protein content (Figure 1C, 1D). We also determined the effect of Wnt5a on CCL2 production by immunofluorescence. As shown by confocal microscopy detection, both Wnt5a transfection and rWnt5a treatment (0.5 µg/ml) increased CCL2 production in macrophages (Figure 1E).

We further determined the expression of chemokine CCL5 and CCL7 which were also involved in macrophage recruitment. ELISA detection showed that macrophages secreted low levels of CCL5 and CCL7 (36±4.2 pg/ml and 24 pg±2.6 pg/ml, respectively), and both Wnt5a transfection and rWnt5a (0.5 µg/ml) treatment had no significant effect on their production (Figure S3A, S3B). Consistent with ELISA detection, real-time PCR also did not reveal a stimulatory effect of Wnt5a on CCL5 and CCL7 mRNA expression (Figure S3C, S3D).

### JNK and NFκB are Involved in Wnt5a-induced CCL2 Upregulation in Macrophages

**Figure 2:**
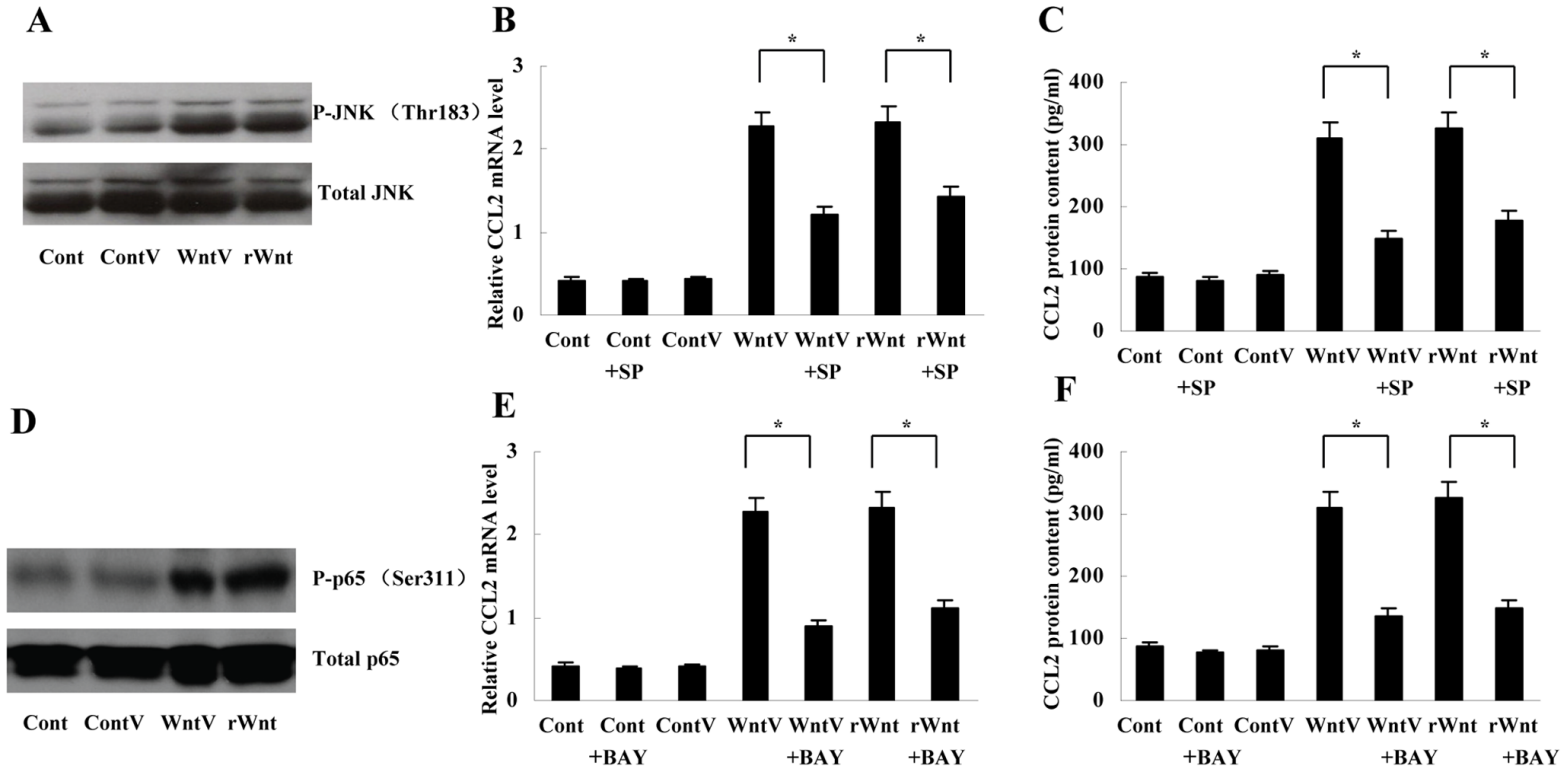
Involvement of JNK and NFκB in Wnt5a-induced CCL2 upregulation in macrophages. (A) Western blot showed that both Wnt5a transfection and rWnt5a treatment (0.5 µg/ml) enhanced the expression of phosphorylated JNK (P-JNK). (B) and (C) Real-time PCR and ELISA showed that JNK inhibitor SP600125 (10 µmol/L) inhibited CCL2 induction by Wnt5a transfection or rWnt5a treatment (0.5 µg/ml). *P<0.01. (D) Western blot showed that both Wnt5a transfection and rWnt5a treatment (0.5 µg/ml) increased the expression of phosphorylated p65 (P-p65). (E) and (F) Real-time PCR and ELISA showed that NFκB inhibitor BAY 11-7082 (10 µmol/L) suppressed CCL2 induction by Wnt5a transfection or rWnt5a treatment (0.5 µg/ml). *P<0.01. Cont, control; ContV, control vector; WntV, Wnt5a vector; rWnt, rWnt5a; SP, SP600125; BAY, BAY 11-7082. Data are expressed as mean±SD, n = 3.

As JNK has been observed to mediate Wnt5a signaling in some types of cells [13], [14], we then investigated its role in Wnt5a-induced CCL2 upregulation in macrophages. Western blot analysis indicated that both Wnt5a transfection and rWnt5a (0.5 µg/ml) treatment stimulated the phosphorylation of JNK (Figure 2A). Moreover, pretreatment with JNK inhibitor SP600125 (10 µmol/L, Sigma) for 45 minutes remarkably inhibited CCL2 induction by Wnt5a expression vector and rWnt5a (0.5 µg/ml) in macrophages (Figure 2B, 2C).

To explore whether NFκB signal was affected by Wnt5a, we examined the expression of phosphorylated pNFκB65 (p-p65) in macrophages with or without Wnt5a treatment. It was found that p-p65 expression was enhanced by both Wnt5a transfection and rWnt5a (0.5 µg/ml) treatment (Figure 2D). NFκB inhibitor BAY 11-7082 (10 µmol/L, Sigma) was subsequently used to pretreat macrophages for 45 minutes, and indeed, CCL2 upregulation induced by Wnt5a expression vector or rWnt5a (0.5 µg/ml) was also inhibited (Figure 2E, 2F).

### Conditioned Medium from Wnt5a-treated Macrophages Induces Macrophage Chemotaxis Mainly Dependent on CCL2

**Figure 3:**
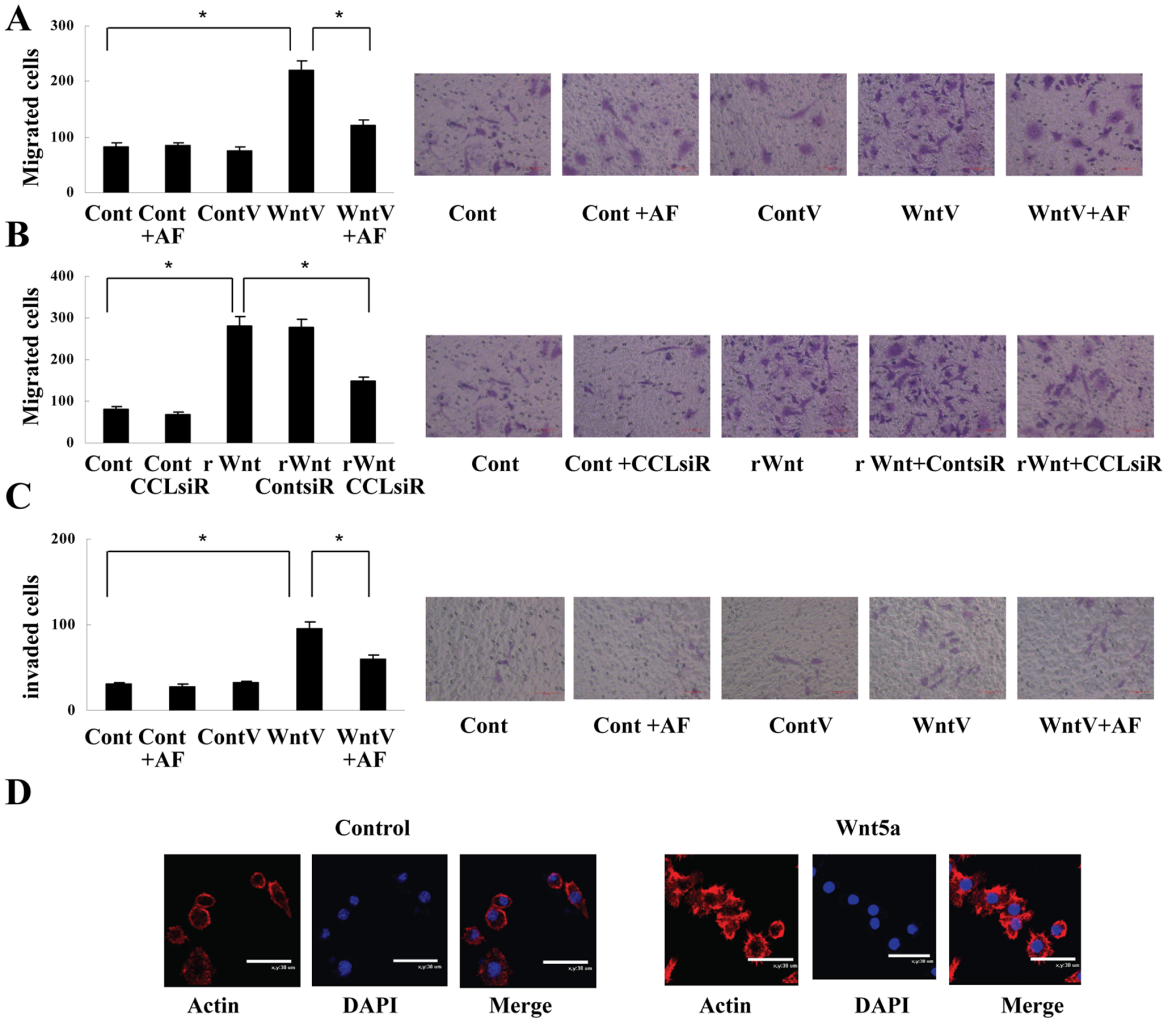
Macrophage chemotaxis induced by conditioned medium from Wnt5a-treated macrophages. (A) Transwell assay showed that conditioned-medium from macrophages treated with Wnt5a expression vector promoted cell migration; the induction in cell migration was suppressed by CCL2 neutralizing antibody AF-479-NA (0.1 µg/ml). *P<0.01. (B) Transwell assay showed that conditioned-medium from macrophages treated with rWnt5a (0.5 µg/ml) increased cell migration; the increased cell migration was abrogated when CCL2 expression was silenced by CCL2 siRNA in macrophages. *P<0.01. (C) Transwell assay showed that conditioned-medium from macrophages treated with Wnt5a expression vector promoted cell invasion; the induction in cell invasion was suppressed by CCL2 neutralizing antibody AF-479-NA (0.1 µg/ml). *P<0.01. (D) Conditioned-medium from macrophages treated with Wnt5a expression vector induced cytoskeletal changes. Cont, control; ContV, control vector; WntV, Wnt5a vector; rWnt, rWnt5a; CCLsiR, CCL2 siRNA; ContsiR, control siRNA; AF, AF-479-NA. Data are expressed as mean±SD, n = 3.

To clarify whether the upregulated CCL2 expression was functional, macrophage migration was analyzed in vitro. It was found that the number of migrated cells from transwell upper chamber increased significantly when conditioned medium from macrophages transfected with Wnt5a expression vector was added to the lower chamber (Figure 3A). To verify the role of CCL2 in the increased cell migration, we added CCL2 neutralizing antibody AF-479-NA (0.1 µg/ml, R&D Systems) into conditioned medium from Wnt5a-treated macrophages. It was observed that the induced cell migration was inhibited significantly (Figure 3A). Moreover, macrophage migration was also enhanced by rWnt5a (0.5 µg/ml), and the increased cell migration was almost abrogated when CCL2 expression in macrophages was silenced by CCL2 siRNA (Figure 3B, Figure S4A). Taken together, these results indicate that Wnt5a treatment chemoattracts macrophages mainly by upregulating CCL2.

Macrophage invasion was assayed with the chamber coated in Matrigel. Similar to migration analysis, macrophage invasion was also increased by conditioned medium from macrophages transfected with Wnt5a, which was also suppressed by AF-479-NA (Figure 3C). In addition, consistent with increased macrophage migration and invasion, significant cytoskeletal changes were observed in macrophages treated with the medium (Figure 3D).

### GEC-derived SFRP5 Inhibits CCL2 Expression and Macrophage Chemotaxis Induced by Wnt5a Treatment

**Figure 4:**
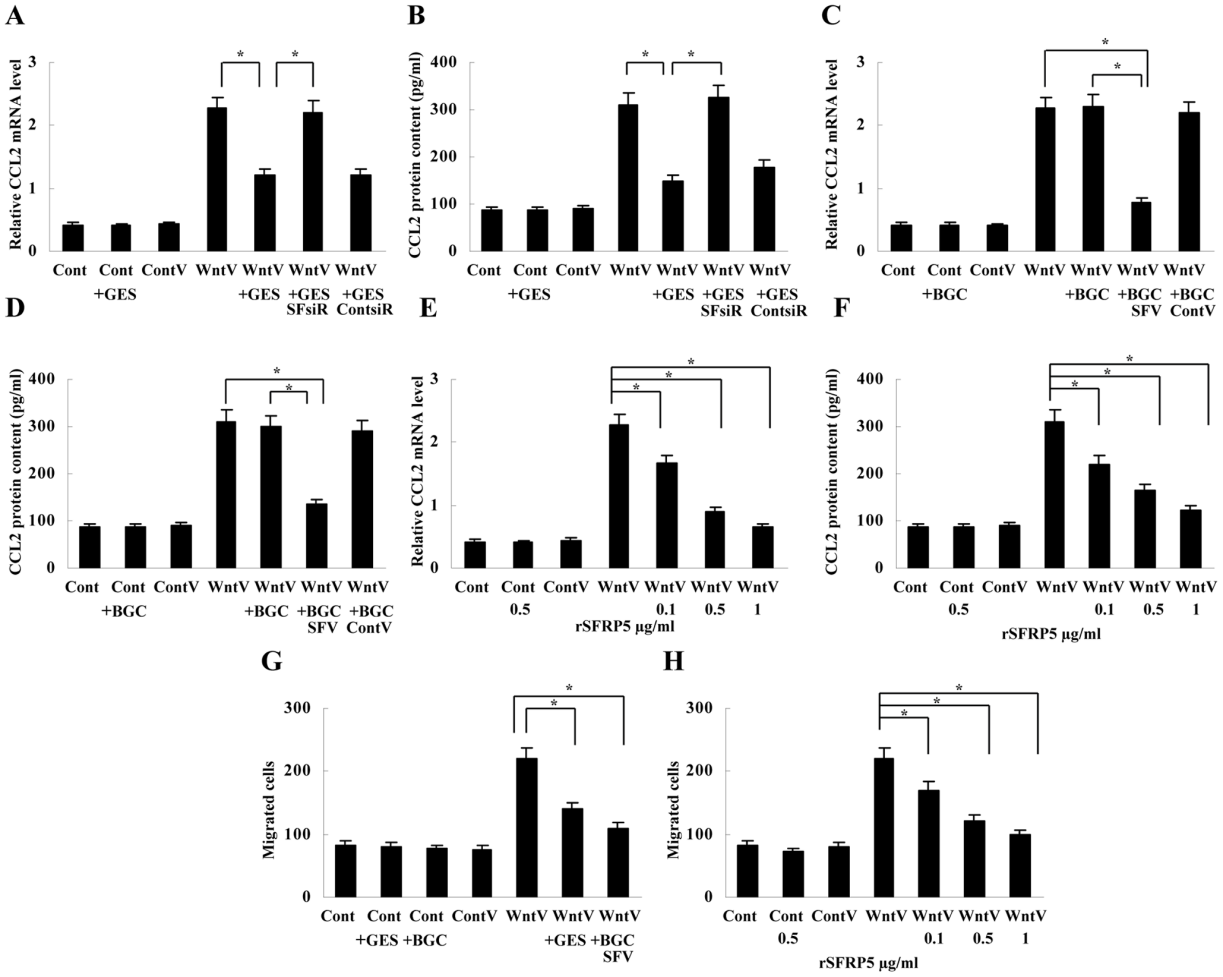
Inhibition of Wnt5a-induced CCL2 expression and macrophage chemotaxis by SFRP5. (A) and (B) Real-time PCR and ELISA showed that conditioned medium from SFRP5-positive GES-1 inhibited Wnt5a-induced CCL2 expression in macrophages; the inhibitory effect of the condition medium was abolished when SFRP5 expression was knocked down in GES-1. *P<0.01. (C) and (D) Real-time PCR and ELISA showed that conditioned medium from SFRP5-transfected BGC-803 suppressed Wnt5a-induced CCL2 expression in macrophages. *P<0.01. (E) and (F) Real-time PCR and ELISA showed that rSFRP5 inhibited CCL2 induction by Wnt5a transfection in a dose-dependent manner. *P<0.01. (G) Transwell assay showed that cell migration induced by conditioned medium from Wnt5a-transfected macrophages was suppressed by co-culture of Wnt5a-transfected macrophages with GES-1 or SFRP5-transfected BGC-803. *P<0.01. (H) Transwell assay showed that cell migration induced by conditioned medium from Wnt5a-transfected macrophages was inhibited by pretreatment of macrophages with rSFRP5. *P<0.01. Cont, control; ContV, control vector; WntV, Wnt5a vector; GES, GES-1; SFsiR, SFRP5 siRNA; ContsiR, control siRNA; BGC, BGC-803; SFV, SFRP5 vector; ContV, control vector. Data are expressed as mean±SD, n = 3.

As SFRP5 has been shown to be an antagonist of Wnt5a, we explored the effect of SFRP5 derived from GECs on Wnt5a-induced CCL2 upregulation in macrophages. Real-time PCR and ELISA analysis indicated that CCL2 induction in macrophages by Wnt5a transfection was inhibited by conditioned medium from SFRP5-positive GES-1 cells. However, the inhibitory effect was abolished when SFRP5 expression was knocked down by SFRP5 siRNA in GES-1 (Figure 4A, 4B, Figure S4B). To further prove the negative effect of SFRP5 on Wnt5a signaling in macrophages, we subsequently treated macrophages with conditioned medium from SFRP5-negative BGC-803 cells with or without SFRP5 expression vector transfection. It was observed that conditioned medium from SFRP5-transfected BGC-803 cells also significantly suppressed Wnt5a-induced CCL2 upregulation, whereas conditioned medium from BGC-803 cells without SFRP5 transfection had no effect on it (Figure 4C, 4D, Figure S4C). Moreover, we also used recombinant SFRP5 (rSFRP5, 0.1 µg/ml, 0.5 µg/ml, 1 µg/ml, respectively, R&D Systems) to treat Wnt5a-transfected macrophage. The detection showed that rSFRP5 inhibited Wnt5a-induced CCL2 expression in a dose-dependent manner (Figure 4E, 4F).

Since Wnt5a-induced CCL2 upregulation was inhibited by SFRP5, we then evaluated the effect of GEC-derived SFRP5 on Wnt5a-induced cell chemotaxis via co-culturing Wnt5a-transfected macrophages with GECs. Migration analysis revealed that conditioned medium from co-cultured macrophages and GES-1 cells or from co-cultured macrophages and SFRP5-transfected BGC-803 cells chemoattracted much less cells than conditioned medium from Wnt5a-transfected macrophages alone (Figure 4F). Furthermore, pretreating macrophages with rSFRP5 (0.1 µg/ml, 0.5 µg/ml, 1 µg/ml, respectively) also suppressed the induced cell migration by conditioned medium from Wnt5a-transfected macrophages (Figure 4G).

### Wnt5a Stimulates Macrophages to Produce Inflammatory Cytokines and COX-2/PGE_2_

**Figure 5:**
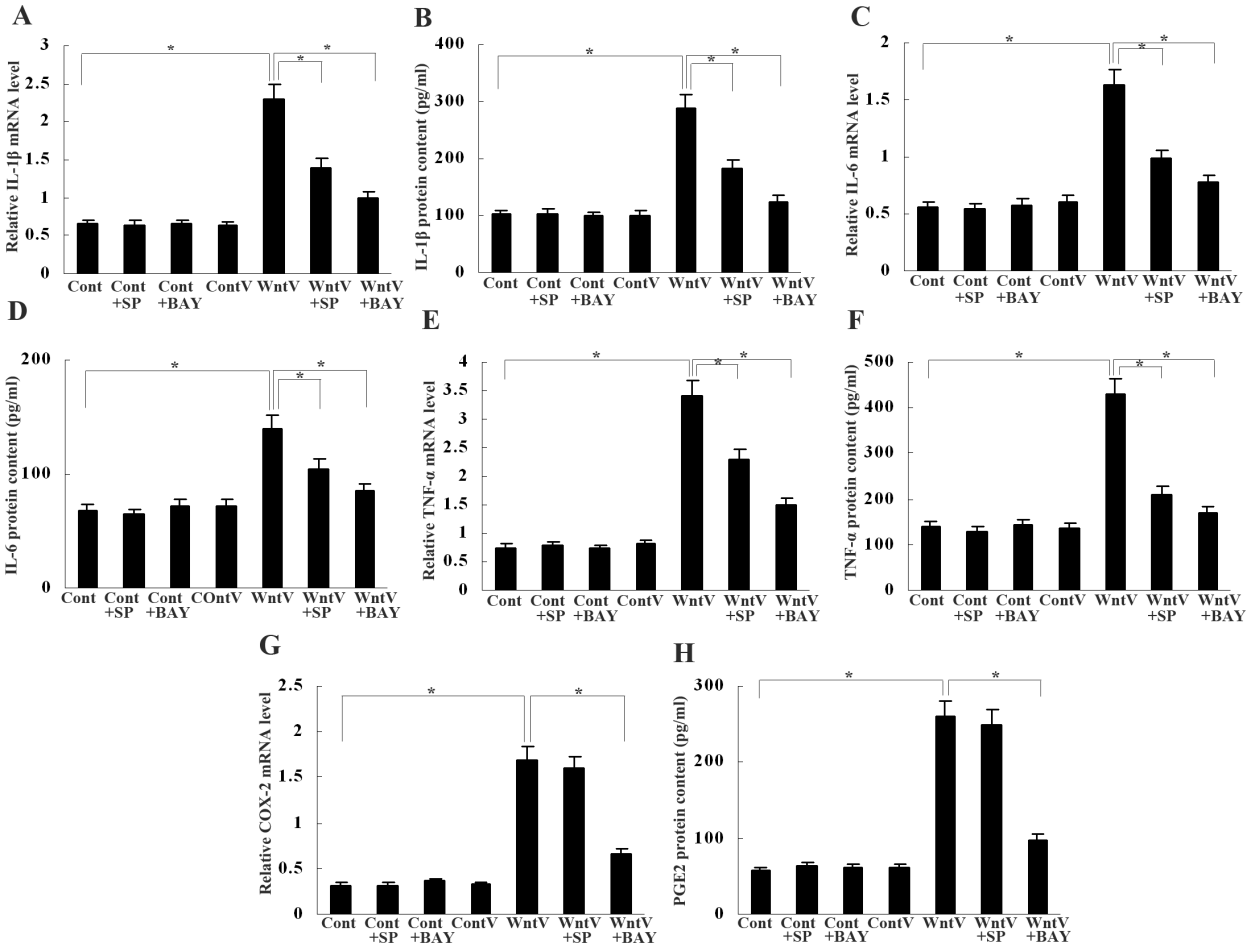
Cytokines and COX-2/PGE_2_ upregulation by Wnt5a in macrophages. (A–F) Real-time PCR and ELISA showed that mRNA expression and protein secretion of IL-1β, IL-6 and TNF-α were upregulated in macrophages by Wnt5a transfection; the upregulation of these cytokines was inhibited by JNK inhibitor SP600125 (10 µmol/L) and NFκB inhibitor BAY 11-7082 (10 µmol/L), respectively. *P<0.01. (G) and (H) Real-time PCR and ELISA showed that COX-2 expression and PGE_2_ production were stimulated by Wnt5a transfection; the induction of COX-2 and PGE_2_ was suppressed by BAY 11-7082 (10 µmol/L), but not by SP600125 (10 µmol/L). *P<0.01. Cont, control; ContV, control vector; WntV, Wnt5a vector; SP, SP600125; BAY, BAY 11-7082. Data are expressed as mean±SD, n = 3.

To assess the effect of Wnt5a on macrophage activation, we examined the production of inflammatory cytokines by macrophages transfected with Wnt5a. Real-time PCR and ELISA detection showed that Wnt5a overexpression promoted mRNA transcription and protein secretion of IL-1β, IL-6 and TNF-α (Figure 5A–5F). Just like CCL2, the upregulation of cytokines induced by Wnt5a transfection in macrophages was also blocked by pretreatment with JNK inhibitor SP600125 (10 µmol/L) or NFκB inhibitor BAY 11-7082 (10 µmol/L) (Figure 5A–5F).

COX-2/PGE_2_ overproduction in gastric mucosa has been well accepted to play an important role in gastric inflammation and carcinogenesis [17]. We then analyzed the effect of Wnt5a on the production of COX-2/PGE_2_ by macrophages. It was found that COX-2 mRNA expression and PGE_2_ secretion were both stimulated by Wnt5a transfection (Figure 5G, 5H). COX-2/PGE_2_ upregulation by Wnt5a was suppressed by BAY 11-7082 (10 µmol/L) as well, however, unlike CCL2 and inflammatory cytokines, it was not affected by SP600125 (Figure 5G, 5H), suggesting JNK signaling was not involved in this process.

### SFRP5 from GECs Suppresses Wnt5a-induced Macrophage Activation

**Figure 6:**
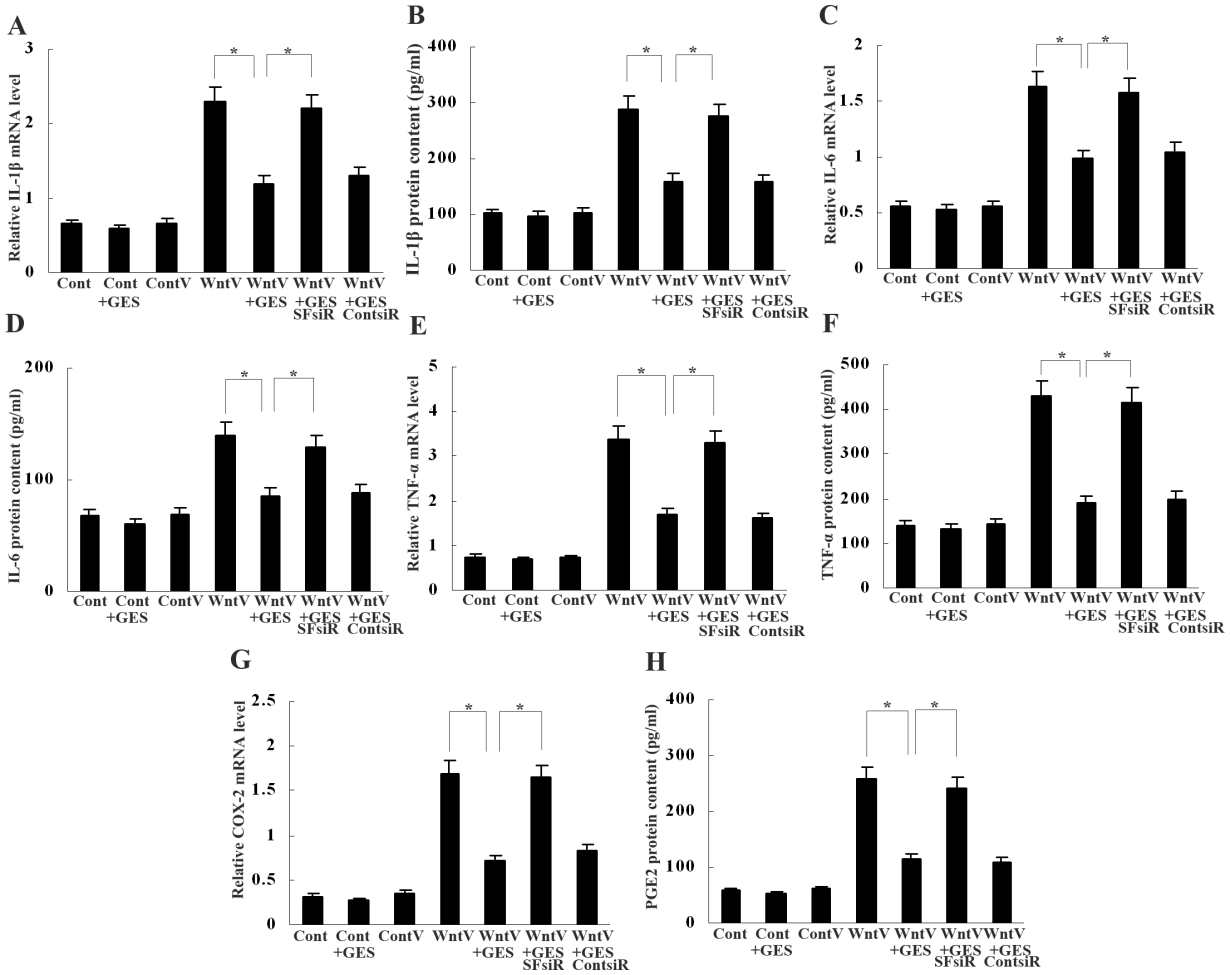
Inhibition of Wnt5a-induced expression of cytokines and COX-2 in macrophages by GES-1 derived SFRP5. (A–H) Real-time PCR and ELISA showed that conditioned medium from SFRP5-positive GES-1 inhibited Wnt5a-induced IL-1β, IL-6, TNF-α and COX-2/PGE_2_ expression in macrophages; the inhibitory effect of the condition medium was abolished when SFRP5 expression was knocked down in GES-1. *P<0.01. Cont, control; ContV, control vector; WntV, Wnt5a vector; GES, GES-1; SFsiR, SFRP5 siRNA; ContsiR, control siRNA. Data are expressed as mean±SD, n = 3.

**Figure 7:**
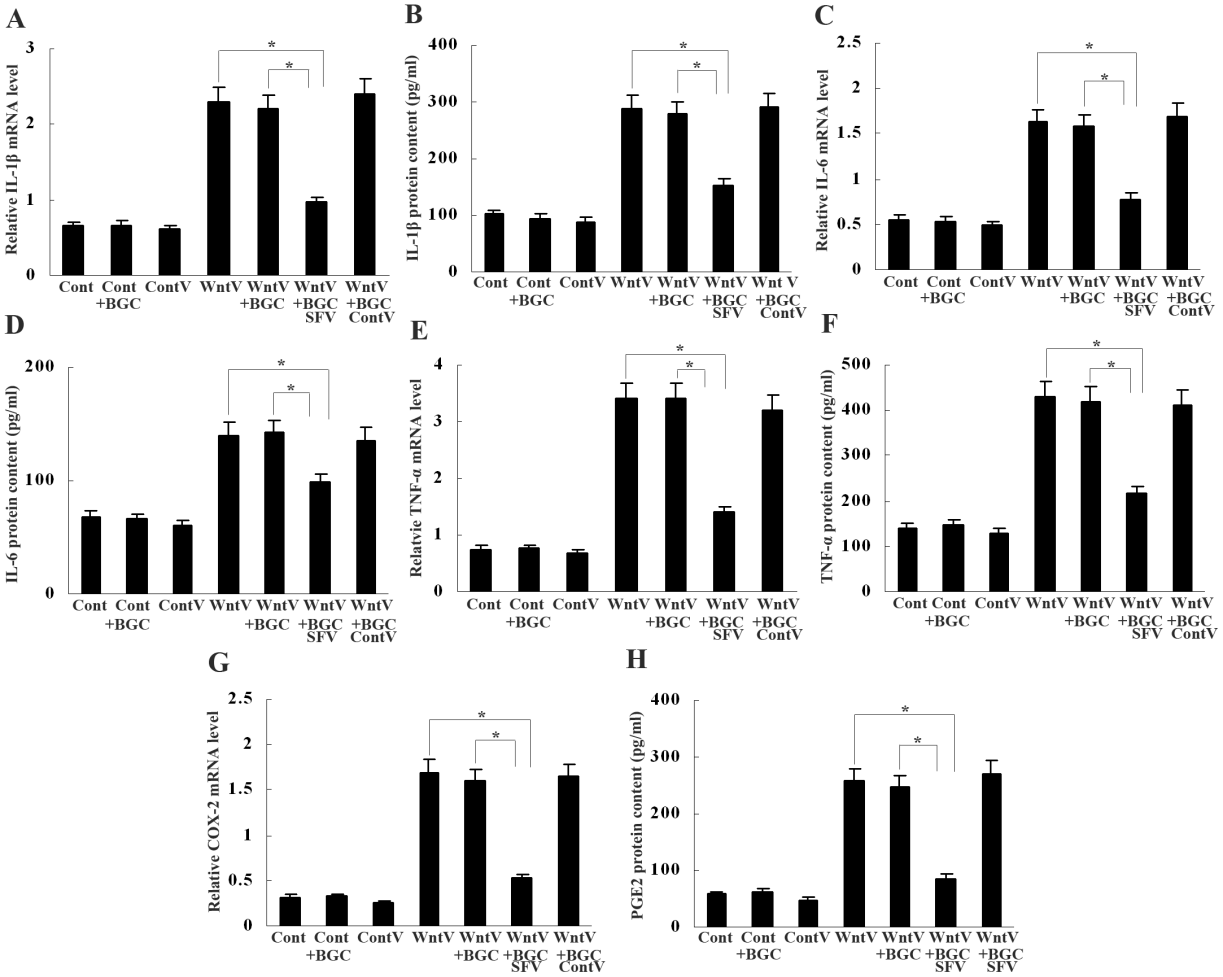
Inhibition of Wnt5a-induced expression of cytokines and COX-2 in macrophages by BGC-803 derived SFRP5. (A–H) Real-time PCR and ELISA showed that conditioned medium from SFRP5-transfected BGC-803 suppressed Wnt5a-induced IL-1β, IL-6, TNF-α and COX-2/PGE2 expression in macrophages. *P<0.01. Cont, control; ContV, control vector; WntV, Wnt5a vector; BGC, BGC-803; SFV, SFRP5 vector; ContV, control vector. Data are expressed as mean±SD, n = 3.

We finally determined the effect of SFRP5 on Wnt5a-induced macrophage activation by treating Wnt5a-transfected macrophages with conditioned medium from GECs. Real-time PCR and ELISA detection revealed that conditioned medium from GES-1 cells weakened IL-1β, IL-6, TNF-α and COX-2 mRNA expression and IL-1β, IL-6, TNF-α and PGE_2_ production by Wnt5a-transfected macrophages, however, conditioned medium from GES-1 with SFRP5 knockdown had no effect on it (Figure 6). In addition, conditioned medium from SFRP5-transfected BGC-803 cells also decreased IL-1β, IL-6, TNF-α and COX-2 mRNA expression and IL-1β, IL-6, TNF-α and PGE_2_ production by Wnt5a-transfected macrophages, whereas conditioned medium from BGC-803 cells without SFRP5 transfection did not (Figure 7). These data indicate that GEC-derived SFRP5 has a negative effect on Wnt5a-induced cytokine and COX-2 expression in macrophages.

## Discussion

Macrophages infiltrating gastric tissues are derived from monocyte in peripheral blood and chemoattrated by locally produced chemokines. Isomoto *et. al.* observed that mucosal CCL2 level was correlated positively with the grades of macrophage infiltration in human gastric carditis [18]. In animal gastric tumor models, it was found that macrophage infiltration was induced by CCL2 transfection [19], but inhibited by anti-CCL2 neutralizing antibody treatment [5]. These studies indicated that CCL2 was responsible for macrophage infiltration in gastric tissues. Importantly, via macrophage recruitment, CCL2 was shown to induce gastric carcinogenesis in orthotopic xenografts [19]. On the other side, macrophage depletion caused by CCL2 inhibition was found to restrict tumor development [5].

CCL2 production in gastric mucosa was shown to be associated with H.pylori infection. It was found that CCL2 level was significantly higher in gastric mucosa of patient with H.pylori infection than those without [18], and H.pylori infection could stimulate GECs to secrete CCL2 in vitro [20]–[22]. These studies suggest that GEC is a source of CCL2 in gastric mucosa. In addition to GECs, macrophage has also been implicated in CCL2 production. CCL2 was expressed in macrophages in H. pylori infected gastritis [20], and stimulation with lipopolysaccharide induced expression of the CCL2 in cultured macrophage RAW264.1 [5]. Moreover, macrophages were also found to produce CCL2 in intestinal mucosa, especially in the condition of inflammation [23].

Our present study explored the effect of Wnt5a on CCL2 secretion by macrophages. Via Wnt5a transfection and rWnt5a treatment, we demonstrated that Wnt5a promoted CCL2 production. As our previous study has revealed that H.pylori can stimulate macrophages to produce and secrete Wnt5a [13], it is reasonable to speculate that Wnt5a is involved in CCL2 induction by H.pylori. Furthermore, we confirmed that macrophage-released CCL2 was functional in macrophage chemotaxis. These data suggest that aberrant Wnt5a production in gastric mucosa may result in macrophage recruitment by upregulating CCL2. Some other chemokines, such as CCL5 and CCL7, were also investigated in the present study. However, our results showed that Wnt5a had no effect on the expression of these two chemokines.

In addition to CCL2, IL-1β, IL-6 and TNF-α were also found to be produced by macrophages in the present study. Upregulation of these inflammatory cytokines by Wnt5a clearly indicates that Wnt5a is a macrophage activator, and plays a role in inflammation. Indeed, Wnt5a has been shown to be involved in some inflammatory processes and diseases [10], [12], [24], [25]. Initially, inflammatory cytokines produced by macrophages may play anti-tumor roles via killing pathogens and tumor cells. However, increasing evidence has demonstrated that persistent existence of these cytokines may finally stimulate carcinogenesis or promote tumor progression. Therefore, our study suggests that Wnt5a is not only involved in inflammation, but also tumor initiation.

It is well established that COX-2/PGE_2_ is implicated in gastric cancer initiation and progression [17]. COX-2 was observed to be overexpressed in gastric dysplasias [26] and gastric cancers [27]. In vitro studies revealed that GEC was a major source of COX-2/PGE_2_, especially under the stimulation of H. pylori [28]. Most importantly, long-term use of COX-2 inhibitor aspirin decreased the mortality of gastric cancer [29], [30]. Taken together, these data suggest that COX-2/PGE_2_ was involved in gastric carcinogenesis. In addition, COX-2 was also shown to be involved in gastric cancer proliferation, angiogenesis, invasion and metastasis [17]. Our study indicates that macrophage is another source of COX-2/PGE_2_ in gastric tissues, and Wnt5a may attribute to gastric cancer initiation via upregulating COX-2/PGE_2_ in macrophages.

**Figure 8:**
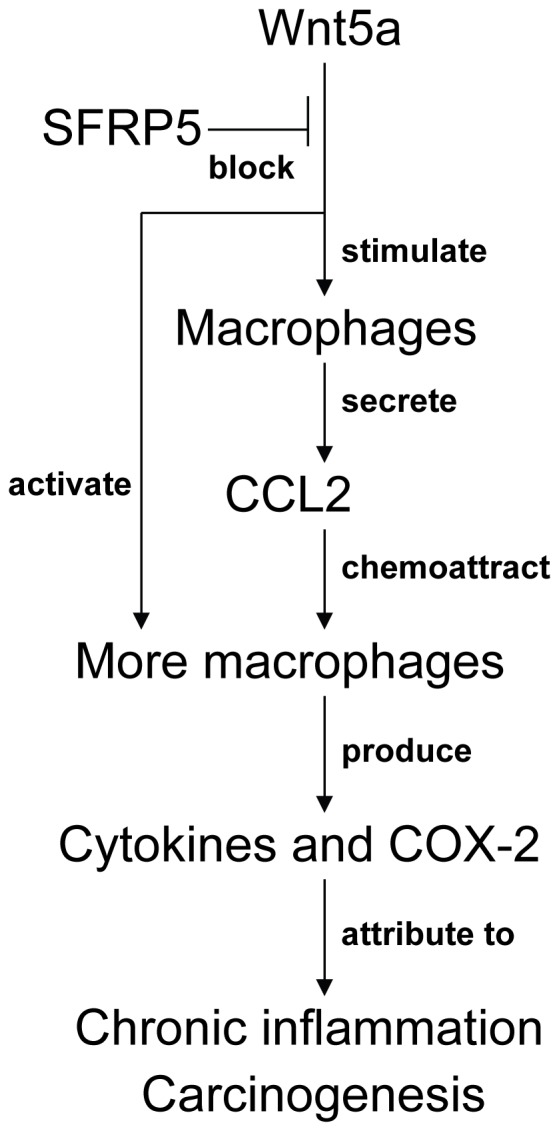
Schema: GEC-derived SFRP5 inhibits Wnt5a-induced macrophage chemotaxis and activation. Wnt5a chemoattracts macrophages by upregulate CCL2 expression, and activates macrophages to secrete cytokines, which are blocked by GEC-derived SFRP5.

The present study revealed that GEC-derived SFRP5 could inhibit Wnt5a-induced macrophage chemotaxis and activation, based on the findings that SFRP5 suppressed the induction of CCL2, cytokines, and COX-2/PGE_2_ by Wnt5a (Figure 8). These data demonstrate that SFRP5 may play a defensive role in Wnt5a-induced gastric inflammation and carcinogenesis. Actually, SFRP5 expression is often downregulated in gastric tissues, which may cause uncontrolled activation Wnt5a signaling. Therefore, Wnt5a overproduction and SFRP5 deficiency in gastric mucosa may together play an important role in gastric cancer initiation.

## Supporting Information

Figure S1 **Effect of Wnt5a on the expression of CCL2 receptor CCR2/4 by Western blot.** (A) Wnt5a was overexpressed in macrophages after transfection with Wnt5a expression vector. (B) Wnt5a transfection had no effect on the expression of CCR2/4. siR, siRNA; V, vector.
(TIF)

Figure S2 **Effect of Wnt5a on the expression of CCL2 mRNA.** Real-time PCR showed that rWnt5a treatment for 12 hours had the strongest effect on CCL2 expression.
(TIF)

Figure S3 **Effect of Wnt5a on CCL5 and CCL7 expression.** Real-time PCR and ELISA showed that neither Wnt5a transfection nor rWnt5a treatment (0.5 µg/ml) had effect on CCL5 and CCL7 expression by macrophages. Control V, transfection with control vector; Wnt5a V, transfection with Wnt5a expression vector. Data are expressed as mean±SD, n = 3.
(TIF)

Figure S4 **CCL2 and SFRP5 knockdown, and SFRP5 overexpression by Western blot.** (A) CCL2 expression was knocked down in macrophages by CCL2 siRNA. (B) SFRP5 expression was knocked down in GES-1 by SFRP5 siRNA. (C) SFRP5 expression was present in BGC-803 after transfection with SFRP5 expression vector.
(TIF)
