# Supplementary figures and images for: GEC-derived SFRP5 Inhibits Wnt5a-Induced Macrophage Chemotaxis and Activation
Source: PLoS One. 2014 Jan 8;9(1):e85058. doi: 10.1371/journal.pone.0085058 (PMC3885681; doi:10.1371/journal.pone.0085058)

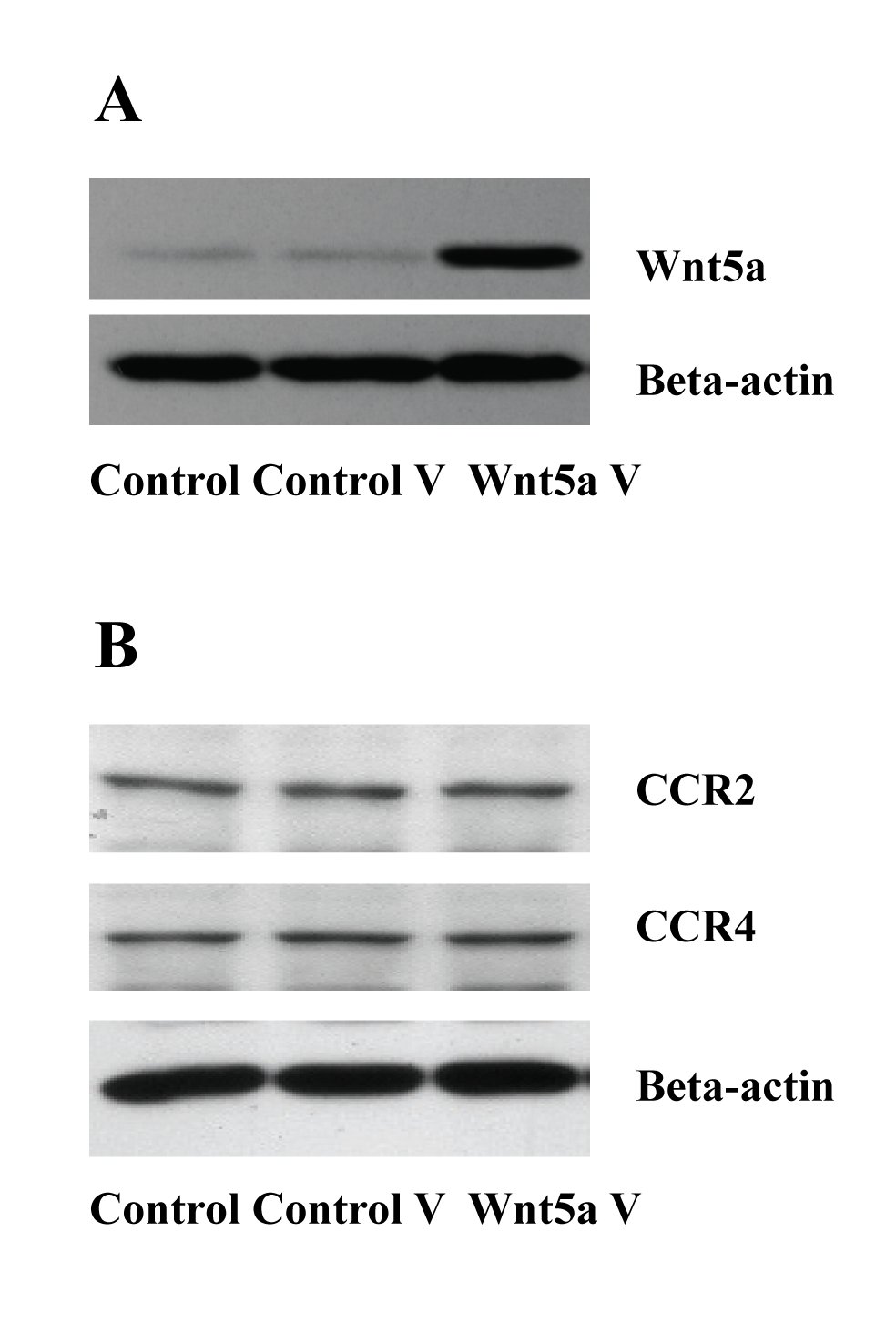

Supplement: Figure S1 — Effect of Wnt5a on the expression of CCL2 receptor CCR2/4 by Western blot. (A) Wnt5a was overexpressed in macrophages after transfection with Wnt5a expression vector. (B) Wnt5a transfection had no effect on the expression of CCR2/4. siR, siRNA; V, vector. (TIF) [file pone.0085058.s001.tif]

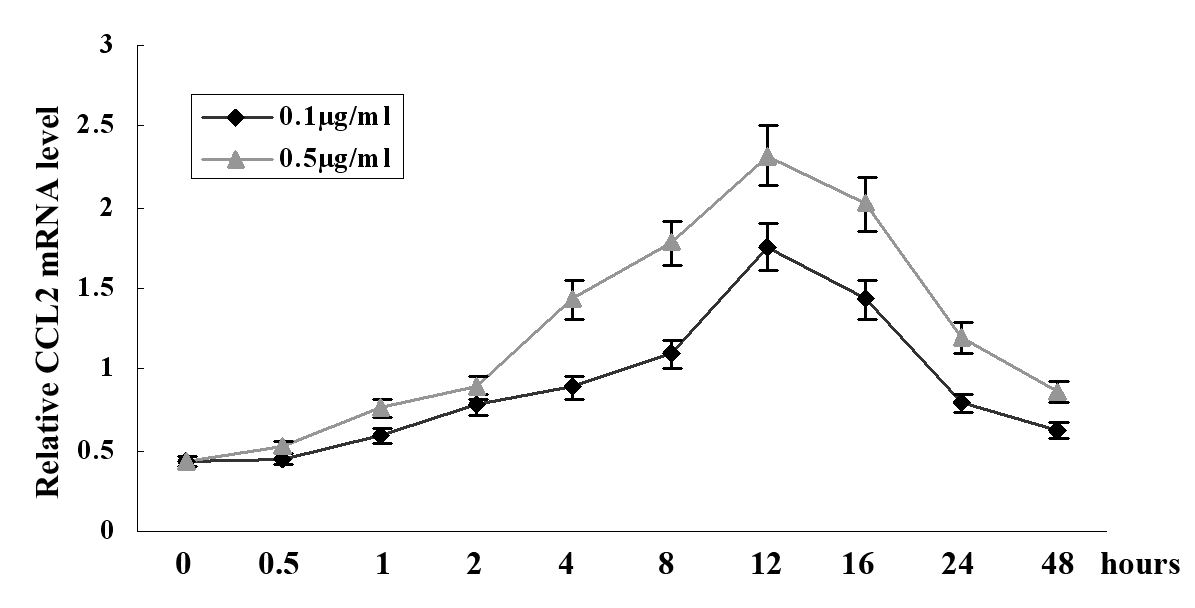

Supplement: Figure S2 — Effect of Wnt5a on the expression of CCL2 mRNA. Real-time PCR showed that rWnt5a treatment for 12 hours had the strongest effect on CCL2 expression. (TIF) [file pone.0085058.s002.tif]

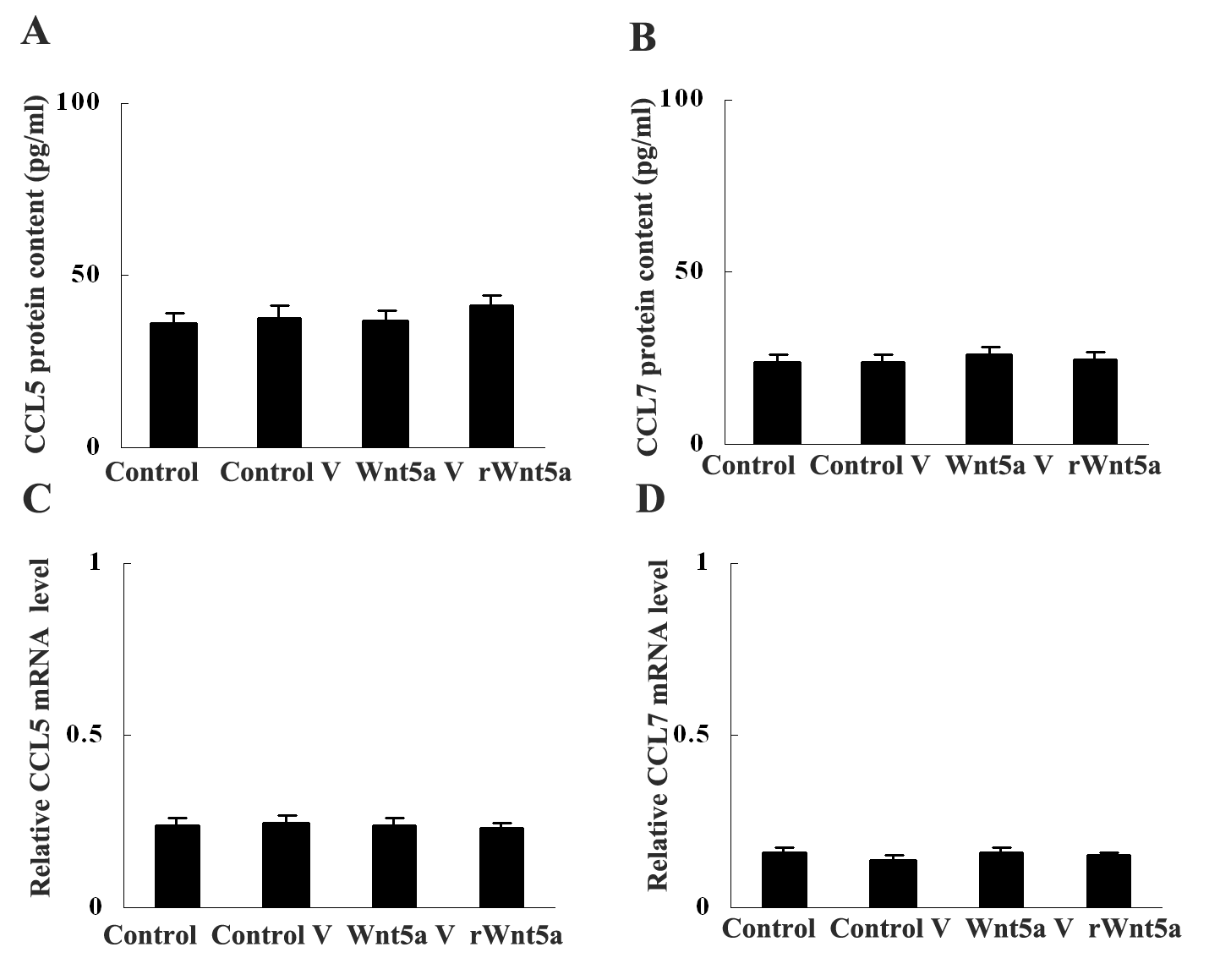

Supplement: Figure S3 — Effect of Wnt5a on CCL5 and CCL7 expression. Real-time PCR and ELISA showed that neither Wnt5a transfection nor rWnt5a treatment (0.5 µg/ml) had effect on CCL5 and CCL7 expression by macrophages. Control V, transfection with control vector; Wnt5a V, transfection with Wnt5a expression vector. Data are expressed as mean±SD, n = 3. (TIF) [file pone.0085058.s003.tif]

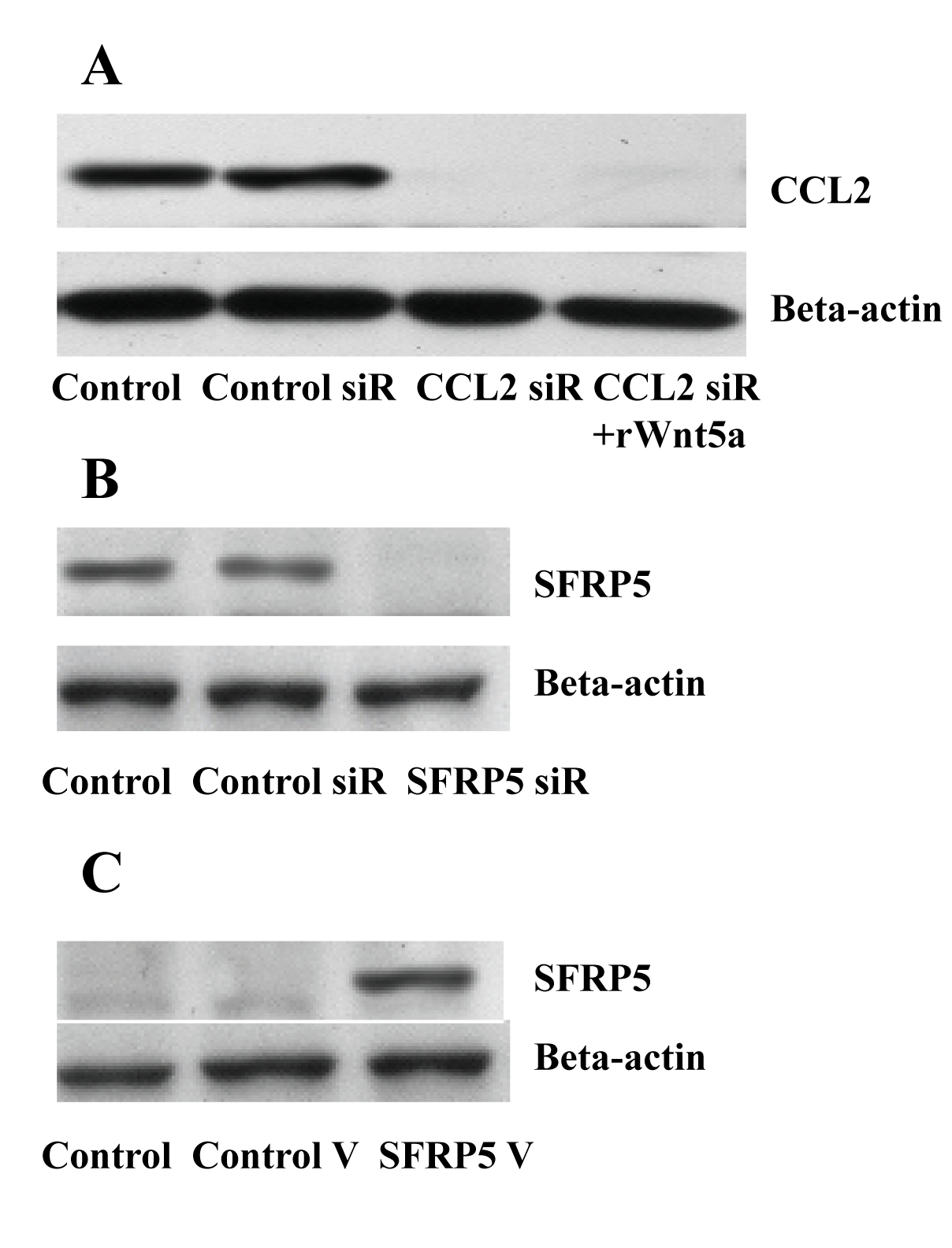

Supplement: Figure S4 — CCL2 and SFRP5 knockdown, and SFRP5 overexpression by Western blot. (A) CCL2 expression was knocked down in macrophages by CCL2 siRNA. (B) SFRP5 expression was knocked down in GES-1 by SFRP5 siRNA. (C) SFRP5 expression was present in BGC-803 after transfection with SFRP5 expression vector. (TIF) [file pone.0085058.s004.tif]
